# Proportions of circulating follicular helper T cells are reduced and correlate with memory B cells in HIV-infected children

**DOI:** 10.1371/journal.pone.0175570

**Published:** 2017-04-26

**Authors:** Daniel M. Muema, Gladys N. Macharia, Babatunde A. Olusola, Amin S. Hassan, Greg W. Fegan, James A. Berkley, Britta C. Urban, Eunice W. Nduati

**Affiliations:** 1 Kenya Medical Research Institute-Wellcome Trust Research Programme, Centre for Geographic Medicine Research-Coast, Kilifi, Kenya; 2 Department of Virology, University of Ibadan, Ibadan, Nigeria; 3 Nuffield Department of Medicine, University of Oxford, Oxford, United Kingdom; 4 Department of Parasitology, Liverpool School of Tropical Medicine, Liverpool, United Kingdom; Jackson Laboratory, UNITED STATES

## Abstract

**Introduction:**

HIV causes defects in memory B cells in children, but the mechanisms of those defects have not been fully elucidated. One possible mechanism is the lack of T-cell help to B cells during immune reactions. However, few studies have assessed the effect of HIV on follicular helper T cells (T_FH_ cells) in children.

**Methods:**

In this study, follicular-homing CD4 T cells and memory B cells were assessed in HIV-infected children and compared with children from the community. CXCR5 and CD45RO were used as markers of follicular-homing T cells and memory T cells, respectively. Memory T_FH_ cells were identified as CD3^+^CD8^-^CD4^+^CXCR5^+^CD45RO^+^PD1^+^. Central memory T cells were identified based on CCR7 expression. Relationship between the proportions of follicular-homing CD4 T cells and memory B cells were determined in multivariable regression models.

**Results:**

Highly viremic HIV-infected children had lower proportions of memory T_FH_ cells when compared with community control children. In multivariable analyses, high proportions of memory T_FH_ cells were associated with increased percentages of resting memory B cells after adjusting for other covariates.

**Conclusion:**

The impact of HIV on follicular helper T cells could influence the accumulation of memory B cells in HIV-infected children.

## Introduction

Even though depletion of CD4 T cells is the hallmark of HIV-induced immune dysfunction, the virus causes many other immunological abnormalities within the CD4 T-cell compartment. CD4 T cells from HIV patients are qualitatively defective, displaying features of aberrant immune activation as depicted by high levels of markers of activation [1]. Paradoxically, they also have impaired responsiveness to stimuli, an observation that has been attributed to the lymphocyte exhaustion that is characterized by up-regulation of inhibitory molecules [2, 3]. HIV is also associated with skewing of the subset-distribution of CD4 T cells. Viremic patients have fewer IL-2 producing central memory CD4 T cells [4]. In addition, active HIV viremia is associated with increased frequencies of follicular helper T cells (T_FH_ cells) in lymphoid tissues, suggesting increased T_FH_ activity [5].

HIV patients also make poor antibody and memory B-cell responses to routine vaccines and common infections [6–14]. The poor memory B-cell responses leave the patients, especially children, prone to repeated infections despite previous exposures and/or immunizations. Considering that one of the major functions of CD4 T cells is to provide help to B cells, the HIV-induced B-cell defects could be due to either depletion of CD4 T cells or HIV-induced qualitative defects in the CD4 T cells. Investigating the effect of HIV on T_FH_ cells, the subset of CD4 T cells that provides help to B cells in germinal centres, is necessary to comprehensively understand the mechanisms by which HIV impairs B-cell responses. Indeed, T_FH_ cells from the lymphoid tissues of HIV patients have been shown to be poor at helping the patients’ B cells in vitro, an effect that has been attributed to increased PD1-PDL1 interaction [15].

Unfortunately, access to lymphoid tissues, the anatomical location of T_FH_ cells, entails performing invasive procedures and is logistically complicated. Attempts have therefore been made to identify counterparts of T_FH_ cells in peripheral circulation. Morita et al identified circulating T_FH_ on the basis of their CXCR5 expression, the marker for follicular homing, and showed that Th2 and Th17 skewing within this subset was associated with active disease in juvenile dermatomyositis [16]. Similarly, Pallikkuth et al used CXCR5 to identify circulating T_FH_ cells and associated their expansion with the magnitude of antibody response against the 2009 H1N1/09 vaccine in HIV patients [17]. Locci et al and Cohen et al described them as CXCR5+CXCR3-PD1+ and CXCR5+PD1+, respectively, and observed an association with eventual development of HIV cross-reactive antibodies [18, 19]. Boswell et al reported that the best B-cell helper capabilities were in the CXCR5^high^CCR6^high^PD1^high^ subset of CD4 T cells, though their frequencies did not correlate with development of cross-reactive neutralizing antibodies [20]. More recently, Schultz et al suggested that IL-21 secretion was the best marker for circulating memory T_FH_ cells [21].

In this study on HIV-infected children, the proportions of circulating T_FH_ cells and other follicular-homing CD4 T cells, and their relationship with memory B cells, were assessed. Considering that most previous studies in HIV used CXCR5 and PD1 to identify circulating T_FH_ cells, the same markers were used here.

## Materials and methods

### Study population

HIV-infected children aged 18 months to 10 years were recruited from the Comprehensive Care and Research Clinic at Kilifi County Hospital in 2012. The children were treated in accordance to the WHO guidelines at the time; those younger than 24 months were put on HAART regardless of their immunological and clinical profile, those between 25 months and 59 months were put on HAART if their CD4 percentages were below 25% or if they were in WHO clinical stages 3 or 4 whereas children above 60 months of age were put on HAART if their CD4 percentages were below 20% or if they were in WHO clinical stages 3 or 4 [22]. Some children were recruited when it was their first time in the clinic (i.e. newly enrolled). The other children were recruited if they had received co-trimoxazole prophylaxis alone or in combination with HAART for at least six months. Viral loads were determined at the International Centre for Reproductive Health in Mombasa using a RT-qPCR test from Agence Nationale de Recherches sur le SIDA with a detection limit of 300 RNA copies/ml [23]. Community control children were recruited from the same community. They did not have fever, malaria parasitemia or any other active infection at the time of collecting the blood. Due to ethical constraints, the community control children were not directly assessed for HIV infection. However, the HIV prevalence among women in the coastal region was 6.1%, and, due to Prevention of Mother To Child Transmission (PMTCT) programs, the HIV prevalence was less than 1% among children in the community [24]. Written informed consent was obtained from the parents or guardians of all children. The study protocol was reviewed and approved by Kenya National Ethical Review Committee of Kenya Medical Research Institute (SSC numbers 1633 and 1131).

### T-cell and B-cell subset determination in PBMCs by flow cytometry

A total of five milliliters of blood were obtained from each child. Plasma and the cellular fractions were separated by centrifugation. Peripheral blood mononuclear cells (PBMCs) were isolated from the cellular fraction by gradient centrifugation and stored in liquid nitrogen at 5 million PBMCs/ml in new born bovine serum containing 10% DMSO. On the day of the assay, PBMCs were thawed and washed in RPMI media containing 10% new born bovine serum, 100 units/ml penicillin, 0.1 mg/ml Streptomycin, 2 mM L-glutamine and 10 mM Hepes buffer.

The following antibodies were used: anti-IgD PE (IADB6), anti-CD19 ECD (J3-119), anti-CD3 ECD (UCHT1) and anti-CD27 PE-Cy5 (1A4CD27) from Beckman Coulter; anti-CD38-PE-Cy7 (HIT2) from eBiosciences; anti-CD8 FITC (DK25) from DAKO; anti-CXCR5 Brilliant Violet 421 (RF8B2), anti-CD4 PE-Cy7 (SK3), anti-CD21 APC (B-ly4) and anti-CD27 Brilliant Violet 421(M-T271) from BD Biosciences; anti-CCR7 APC (G043H7), anti-CD45RO APC-Cy7 (UCHL1) and anti-CD10 PE-Cy5 (HI10a) from Biolegend.

PBMCs were stained with various cocktails of the surface markers. At least 80,000 events were acquired on a nine-colour Cyan ADP flow cytometer (Beckman Coulter). Analyses and determination of different subsets were done in FlowJo software version 10 (TreeStar inc, Flowjo Africa Scheme).

Total follicular-homing CD4 T cells were identified based on the expression of CXCR5 i.e. CD3^+^CD8^-^CD4^+^CXCR5^+^. Since circulating follicular helper cells are expected to be antigen-experienced, the proportions of memory follicular-homing CD4 T cells were also determined based on the additional expression of CD45RO, the marker of memory in T cells i.e. CD3^+^CD8^-^CD4^+^CXCR5^+^CD45RO^+^. Since some studies have shown that the B-cell helper capabilities are enriched in the PD1^+^CXCR5^+^ memory CD4 T cells, the proportions of CD3^+^CD8^-^CD4^+^CXCR5^+^CD45RO^+^PD1^+^ were also determined, here referred as memory follicular helper CD4 T cells (memory T_FH_ cells). Within the memory compartments, central memory CD4 T cells were determined as CCR7^+^ cells, whereas effector memory CD4 T cells were determined as CCR7^-^ cells.

Total resting memory B cells were identified as CD19^+^CD27^+^CD21^+^CD10^-^CD38^-/+^ as described in previous studies [13, 25]. Further analyses were done to identify IgD^+^ (unswitched) and IgD^-^ (switched) resting memory B cells.

### Statistical analyses

Active HIV viremia has been previously associated with B-cell defects [9, 13, 14, 26]. Therefore, the HIV-infected children were stratified into high viremia and low viremia groups based on a cut off of 5000 copies/ml, similar to our previous study and the WHO definition of treatment failure as at the time of enrollment of the children [13, 22].

Comparisons between different groups of children were done using the Mann-Whitney test (Wilcoxon rank sum test). Correlations were done using Spearman’s rank-order correlation. Multivariable quantile regression analyses were done to assess independent relationships between various parameters. P values were considered significant if they were less than 0.05. STATA version 13 (STATA corporation) was used for all analyses.

## Results

### Characteristics of the study population

The population characteristics of the participants are shown in Table 1. A total of 52 children were recruited into the study of whom 36 were HIV-infected and 16 were community control children. Of the HIV-infected children, 15 had high viremia. Median viral loads were 4.6 (IQR, 4.3–5.0) log_10_RNA copies/ml in the high viremia group and 1.9 (IQR, 0.0–3.1) log_10_RNA copies/ml in the low viremia group (P<0.01). Nine of the high viremia children and 20 of the low viremia children were on HAART (P<0.01). CD4 T percentages data were available for 28 of the 36 HIV-infected children. Full blood counts data were available for 32 of the 36 HIV-infected children and all 16 community controls. The high viremia group had fewer monocytes when compared with the community controls (median 0.40 [IQR, 0.29–0.53] x 10^3^ cells/μL versus median 0.49 [IQR, 0.41–0.60] x 10^3^ cells/μL, P = 0.04). Interestingly, the low viremia group had fewer eosinophils when compared with both high viremia group (median 0.19 [IQR, 0.11–0.34] x 10^3^ cells/μL versus median 0.53 [IQR, 0.15–1.02] x 10^3^ cells/μL, P = 0.01) and community controls (median 0.19 [IQR, 0.11–0.34] x 10^3^ cells/μL versus median 0.49 [IQR, 0.30–0.68] x 10^3^ cells/μL, P<0.01). The high viremia group had lower hemoglobin levels when compared with community controls (median 10 [IQR, 9–11] g/dL versus median 11 [IQR, 11–12] g/dL, P = 0.02).

**Table 1:** Characteristics of the study population.

	High viremia (HV)	Low viremia (LV)	Community controls (CC)	Normal range	P value		
					HV vs LV	HVvs CC	LV vs CC
N	15	21	16	N/A			
Age (years)	5.9 (3.8–6.9)	5.3 (2.6–8.0)	5.8 (5.0–6.6)	N/A	0.96	0.75	0.69
% CD4+ T cells	22.0 (16.7–34.4)	30.0 (20.7–36.2)	N/D		0.31	N/A	N/A
HIV log_10_ RNA copies/ml	4.6 (4.3–5.0)	1.9 (0.0–3.1)	N/A	N/A	**<0.01**	N/A	N/A
% on HAART (n)	60% (9)	95% (20)	N/A	N/A	**<0.01**	N/A	N/A
Lymphocytes (x 10^3^/μL)	3.8 (2.3–4.2)	3.6 (3.1–4.5)	3.4 (3.1–3.9)	1.7–7.6	0.65	0.63	0.43
Monocytes (x 10^3^/μL)	0.40 (0.29–0.53)	0.52 (0.34–0.66)	0.49 (0.41–0.60)	0.3–1.5	0.22	**0.04**	0.83
Neutrophils (x 10^3^/μL)	2.0 (1.3–2.5)	2.6 (1.6–3.9)	2.3 (2.1–3.6)	1.2–5.5	0.23	0.13	0.79
Eosinophils (x 10^3^/μL)	0.53 (0.15–1.02)	0.19 (0.11–0.34)	0.49 (0.30–0.68)	0.1–1.3	**0.01**	0.93	**<0.01**
Platelets (x 10^3^/μL)	349 (293–449)	365 (292–459)	370 (278–444)	159–564	0.98	0.93	0.76
Hemoglobin (g/dL)	10 (9–11)	11 (10–11)	11 (11–12)	8.2–12.7	0.15	**0.02**	0.11

Values shown for age, % CD4+ T cells, HIV log_10_ RNA copies/ml, lymphocytes, monocytes, neutrophils, eosinophils, platelets and hemoglobin are medians and interquartile ranges. Values under the normal range column refer to predetermined hematology reference values of normal children in Kilifi county. % CD4+ T cells refer to frequencies of CD4 T cells as percentage of total lymphocytes. Statistical tests used: Wilcoxon rank sum test was used for age, % CD4+ T cells, HIV log_10_ RNA copies/ml, lymphocytes, monocytes, neutrophils, eosinophils, platelets and hemoglobin. Chi-squared test was used for % on HAART. N/D—Not determined. N/A—Not applicable.

### HIV-infected children have reduced proportions of circulating follicular-homing CD4 T cells

**Fig 1:**
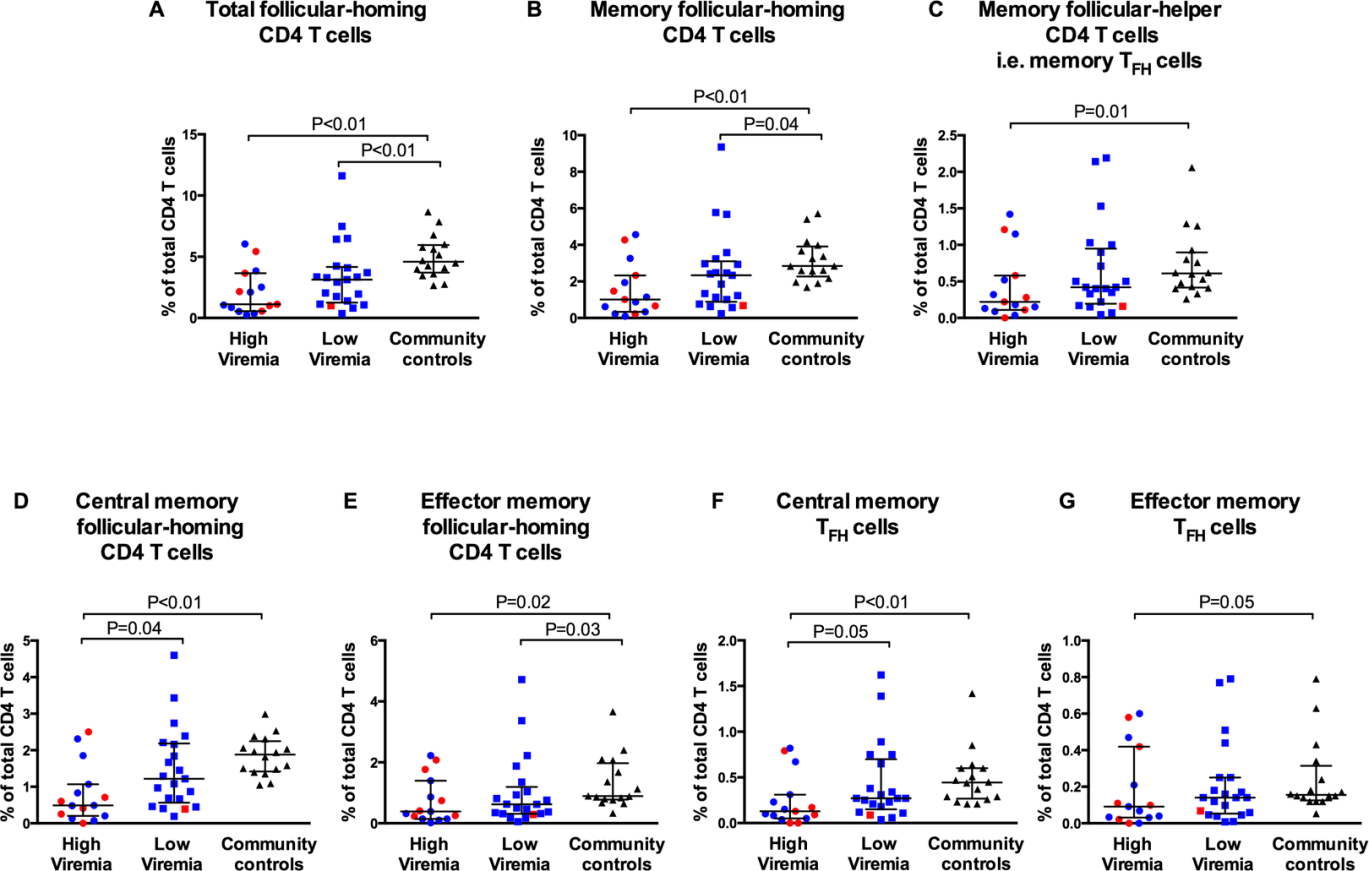
HIV infection is associated with low proportions of circulating follicular helper T cells (T_FH_ cells). Proportions of (A) total follicular-homing CD4 T cells (CXCR5^+^CD4^+^), (B) memory follicular-homing CD4 T cells (CXCR5^+^CD45RO^+^CD4^+^), (C) memory T_FH_ cells (CXCR5^+^CD45RO^+^PD1^+^CD4^+^), (D) central memory follicular-homing CD4 T cells (CXCR5^+^CD45RO^+^CCR7^+^CD4^+^), (E) effector memory follicular-homing CD4 T cells (CXCR5^+^CD45RO^+^CCR7^-^CD4^+^), (F) central memory T_FH_ cells (CXCR5^+^CD45RO^+^PD1^+^CCR7^+^CD4^+^) and (G) effector memory T_FH_ cells (CXCR5^+^CD45RO^+^PD1^+^CCR7^-^CD4^+^) as percentages of total CD4 T cells. Each symbol represents a child. High viremia, ≥5000 RNA copies/ml; Low viremia, <5000 RNA copies/ml. Horizontal lines and error bars represent median, 25^th^ and 75^th^ percentiles. Red symbols represent HIV-infected children who were not on HAART. Blue symbols represent HAART-treated children. Statistical test: Wilcoxon’s rank sum test.

Median proportions of total follicular-homing CD4 T cells (CD3^+^CD8^-^CD4^+^CXCR5^+^), memory follicular-homing CD4 T cells (CD3^+^CD8^-^CD4^+^CXCR5^+^CD45RO^+^) and memory T_FH_ cells (CD3^+^CD8^-^CD4^+^CXCR5^+^CD45RO^+^PD1^+^) were lower in both HIV-infected groups when compared with community controls, except for memory T_FH_ cells in the low viremia group (Fig 1A–1C). The high viremia group also had significantly lower frequencies of central memory follicular-homing CD4 T cells and central memory T_FH_ cells when compared with the low viremia and community control groups (Fig 1D and 1F). In addition, the two HIV-infected groups had lower frequencies of effector memory follicular-homing CD4 T cells when compared with the community controls group (Fig 1E). In the memory T_FH_ compartment, the high viremia group, but not the low viremia group, had lower frequencies of effector memory T_FH_ cells when compared with the community controls group (Fig 1G).

**Fig 2:**
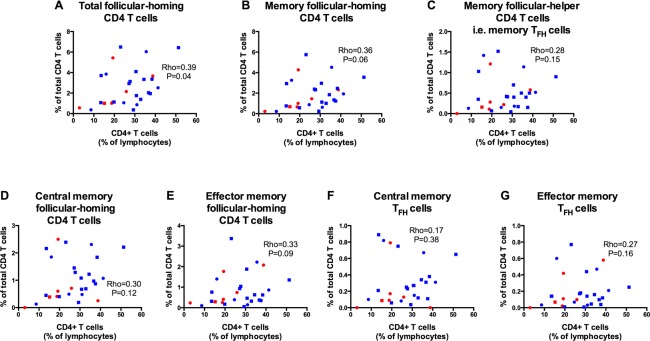
In the HIV-infected cohort, proportions of total follicular-homing CD4 T cells are directly correlated with CD4 percentages. Correlation analyses between CD4 T cells (as percentage of total lymphocytes) and: (A) total follicular-homing CD4 T cells (CXCR5^+^CD4^+^), (B) memory follicular-homing CD4 T cells (CXCR5^+^CD45RO^+^CD4^+^), (C) memory T_FH_ cells (CXCR5^+^CD45RO^+^PD1^+^CD4^+^), (D) central memory follicular-homing CD4 T cells (CXCR5^+^CD45RO^+^CCR7^+^CD4^+^), (E) effector memory follicular-homing CD4 T cells (CXCR5^+^CD45RO^+^CCR7^-^CD4^+^), (F) central memory T_FH_ cells (CXCR5^+^CD45RO^+^PD1^+^CCR7^+^CD4^+^) and (G) effector memory T_FH_ cells (CXCR5^+^CD45RO^+^PD1^+^CCR7^-^CD4^+^). Circles represent highly viremic children and squares represent lowly viremic children. Red symbols represent HAART-naïve children and blue symbols represent HAART-treated children. Statistical test: Spearman’s rank-order correlation.

Among all HIV-infected children, there was a direct correlation between total CD4 percentages and total follicular-homing CD4 T cells. A similar but non-significant trend was observed between total CD4 percentages and memory follicular-homing CD4 T cells (P = 0.06) but not memory T_FH_ cells (Fig 2A–2C). We also observed a trend towards direct correlation between total CD4 percentages and effector memory follicular-homing CD4 T cells (P = 0.09), but no relationship with other memory follicular-homing subsets (Fig 2D–2G).

### Low proportions of memory T_FH_ cells are independent predictors of reduced proportions of resting memory B cells in HIV-infected children

**Fig 3:**
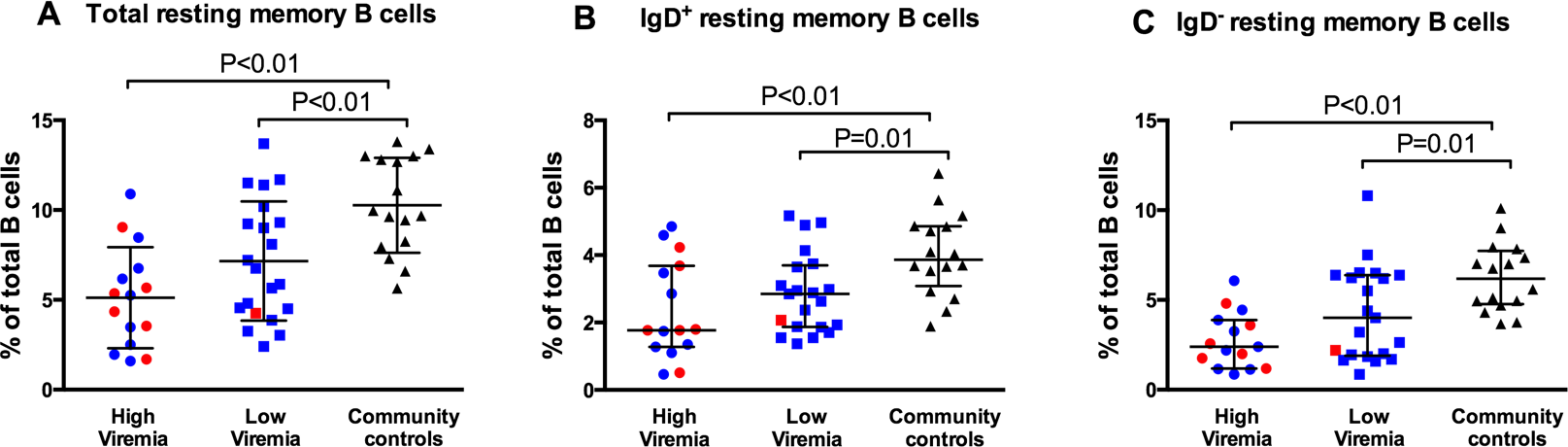
HIV-infected children have reduced proportions of resting memory B cells. Proportions of (A) total resting memory B cells, (B) IgD^+^ resting memory B cells and (C) IgD^-^ resting memory B cells as percentages of total B cells. Each symbol represents a child. High viremia, ≥5000 RNA copies/ml; Low viremia, <5000 RNA copies/ml. Horizontal lines and error bars represent median, 25^th^ and 75^th^ percentiles. Red symbols represent HIV-infected children who were not on HAART. Blue symbols represent HAART-treated children. Statistical test: Wilcoxon’s rank sum test.

To assess the relationship between memory follicular-homing CD4 T cells and memory B cells, the proportions of resting memory B cells were determined in accordance to previous studies [13, 14, 25]. Similar to the previous reports, when compared with community controls, both high viremia and low viremia groups had lower proportions of total resting memory B cells (P<0.01 in both cases), IgD^+^ (Unswitched) resting memory B cells (P<0.01 and P = 0.01, respectively) and IgD^-^ (switched) resting memory B cells (P<0.01 and P = 0.01, respectively) (Fig 3A–3C) [9, 11, 13, 14, 27].

Some reports have suggested that CXCR5+PD1+ memory CD4 T cells are the main predictors of B-cell function with regard to the development of broadly neutralizing antibodies in HIV patients [18, 19]. Therefore, the association between memory T_FH_ cells (CD3^+^CD8^-^CD4^+^CXCR5^+^CD45RO^+^PD1^+^) and resting memory B cells was assessed in multivariable quantile regression analyses. When the independent predictions of resting memory B-cell subsets by HIV infection, age, memory T_FH_ cells, high viremia, interaction of age with high viremia and HAART were assessed, lower proportions of memory T_FH_ cells were predictors of lower proportions of total and IgD^-^ resting memory B cells. Being older was a predictor of higher proportions of IgD^-^ resting memory B cells (Table 2). The covariates HIV infection, age, high viremia, interaction of age with high viremia and HAART had been identified in a previous larger study [13].

**Table 2:** Estimated change (beta coefficients) in frequencies of memory B-cell subsets with variations in age, HIV status, proportions of memory T_FH_ cells, viremia and HAART in multivariable regression.

	Beta Coefficient (Standard Error)	P value
**Total resting memory**		
Age (Years)	0.3 (0.3)	0.30
HIV infection	-3.9 (2.2)	0.08
Memory T_FH_ cells	3.2 (1.1)	**<0.01**
High viremia	-1.1 (3.0)	0.72
Age*high viremia	0.0 (0.5)	0.96
HAART	0.6 (1.9)	0.76
**IgD+ resting memory**		
Age (Years)	-0.1 (0.1)	0.46
HIV infection	-1.4 (1.1)	0.22
Memory T_FH_ cells	0.9 (0.5)	0.13
High viremia	-1.9 (1.6)	0.23
Age*high viremia	0.3 (0.3)	0.27
HAART	0.4 (1.0)	0.68
**IgD- resting memory**		
Age (Years)	0.5 (0.2)	**0.02**
HIV infection	-2.0 (1.5)	0.20
Memory T_FH_ cells	2.0 (0.7)	**0.01**
High viremia	0.8 (2.1)	0.71
Age*high viremia	-0.3 (0.3)	0.39
HAART	0.2 (1.3)	0.89

Each memory B-cell subset was independently predicted in a quantile regression model incorporating the reported variables. Age*high viremia is an interaction term for age and level of viremia. P values <0.05 were considered significant. Significant results are in bold text.

In additional analyses on the predictive ability of other subsets within the follicular-homing CD4 T cells compartment, lower proportions of total follicular-homing CD4 T cells were predictors for lower proportions of total and IgD^+^ resting memory B cells (P<0.01 and P = 0.02, respectively). Similarly, low proportions of memory follicular-homing CD4 T cells were predictors for lower proportions of total and IgD^+^ resting memory B cells (P<0.01 and P = 0.02, respectively). Low proportions of central memory follicular-homing CD4 T cells were predictors for low proportions of total and IgD^-^ resting memory B cells (P<0.01 and P = 0.03, respectively) whereas low proportions of effector memory follicular-homing CD4 T cells were predictors for low proportions of total and IgD^+^ resting memory B cells (P = 0.02 and P<0.01, respectively). Low proportions of central memory T_FH_ cells were predictors for low proportions of total and IgD^-^ resting memory B cells (P = 0.02 in both cases) whereas low proportions of effector memory T_FH_ cells were predictors for low proportions of total and IgD^+^ resting memory B cells (P<0.01 in both cases) (Data not shown).

## Discussion

Since antibodies have proven to play a critical role in vaccine mediated immunity against many infections, it would be important to understand the factors that determine the magnitude and quality of B-cell responses. Defects in the B-cell compartment leave HIV patients exposed to many opportunistic infections that would otherwise be controlled by a normal antibody response [6, 7, 10, 12]. This problem is particularly significant in HIV-infected children, whose immature immune system develops in a background of HIV infection, leading to faster disease progression and probably more severe defects [28, 29]. Understanding the mechanisms by which HIV causes B-cell defects could inform interventions to improve antibody responses in HIV-infected children.

T_FH_ cells are the distinct CD4 T cell subset that provides help to B cells inside B-cell follicles in lymphoid tissues [30]. The recent identification of memory circulating counterparts of T_FH_ cells in blood has enabled studies that evaluate how HIV-induced defects on the T_FH_ compartment affect B-cell responses [17–19, 27].

In the current study, we show that the proportions of circulating follicular-homing CD4 T cells are lower in HIV-infected children when compared to community control children, similar to a previous report [27]. HIV preferentially replicates in T_FH_ cells in lymphoid tissues, probably leading to their preferential destruction, a phenomenon that could spill over into the circulating memory T_FH_ cells compartment [31]. Furthermore, B cells play a major role in reciprocally maintaining T_FH_ cells via co-stimulatory signals, and the detrimental effect of HIV on the B-cell compartment could indirectly impact the T_FH_ compartment [15]. Increased numbers of T_FH_ cells have been observed in lymphoid tissues of HIV patients, suggesting that depletion in peripheral blood could alternatively be due to, at least in part, increased trafficking into lymphoid organs [5, 32].

Notably, low proportions of memory T_FH_ cells in HIV-infected children were associated with reduced proportions of total and IgD^-^ (class-switched) resting memory B cells in multivariable analyses after adjusting for other covariates. Previous reports have shown a similar link between frequencies of circulating memory T_FH_ cells and the quality of B-cell responses [17–19, 27]. T_FH_ cells in lymphoid tissues are important for the formation of germinal centers, the histological sites where long-lived plasma cells and memory B cells are generated during immune reactions. They also provide important contact and soluble signals that drive B-cell affinity maturation and class switch recombination, processes that determine the quality of the B-cell response [30]. The observed paucity of circulating memory T_FH_ cells implies that HIV-infected children have impaired T_FH_ activity resulting into the observed impaired generation of memory B cells. The reduced B-cell helper activity could be exacerbated by other qualitative defects in addition to these quantitative defects. For instance, memory T_FH_ cells from HIV-infected children could have altered differentiation patterns into less functional effector phenotypes. In this study, viremic HIV-infected children had fewer proportions of both central and effector memory T_FH_ cells, suggesting that the paucity of central memory T_FH_ cells was not due to differentiation into effector memory T_FH_ cells. However, T_FH_ cells from HIV-infected children have previously been shown to be defective at secreting IL-4, a cytokine that constitutes T-cell help to B cells and is required for maintenance of germinal centers, thus influencing the development of memory B cells [27, 30]. It is not known if the expression of surface molecules that mediate T-cell to B-cell interactions is altered in various subsets of follicular-homing CD4 T cells in HIV-infected children. Additional studies are required to assess such possibilities. Of note, the observed relationship between memory T_FH_ cells and memory B cells does not rule out the possibility of direct inhibition of B-cell responses by virion factors as suggested by some studies [33, 34].

Notably, the low viremia children also had reduced proportions of some of the subsets within follicular-homing CD4 T-cell compartment when compared with community controls. This observation implies that controlling viremia does not fully restore the memory T_FH_ compartment and that the HAART-treated children would need to rebuild the memory T_FH_ compartment through re-exposures to the various antigens. As such, re-immunization programmes could be beneficial to HAART-treated HIV-infected children.

Due to sample limitation, our study could not assess antigen-specific responses. It would be interesting to determine if similar results will be obtained with regard to antigen-specific memory T_FH_ cells and antigen specific B cells in HIV-infected children, and whether such responses predict the quality of antibody responses upon revaccination. Nevertheless, the data suggest that designing interventions that modulate T_FH_ cells could lead to better B-cell responses in HIV-infected children.

## Supporting information

S1 Dataset (DTA)

S1 Fig Representative flow-cytometry plots showing the gating strategy for identifying various subsets of follicular-homing CD4 T cells. (PDF)

S2 Fig Representative flow-cytometry plots showing the gating strategy for identifying resting memory B cells. (PDF)
