# Supplementary material for: Proportions of circulating follicular helper T cells are reduced and correlate with memory B cells in HIV-infected children
Source: PLoS One. 2017 Apr 26;12(4):e0175570. doi: 10.1371/journal.pone.0175570 (PMC5405965; doi:10.1371/journal.pone.0175570)

Representative flow-cytometry plots showing the gating strategy for identifying resting memory B cells

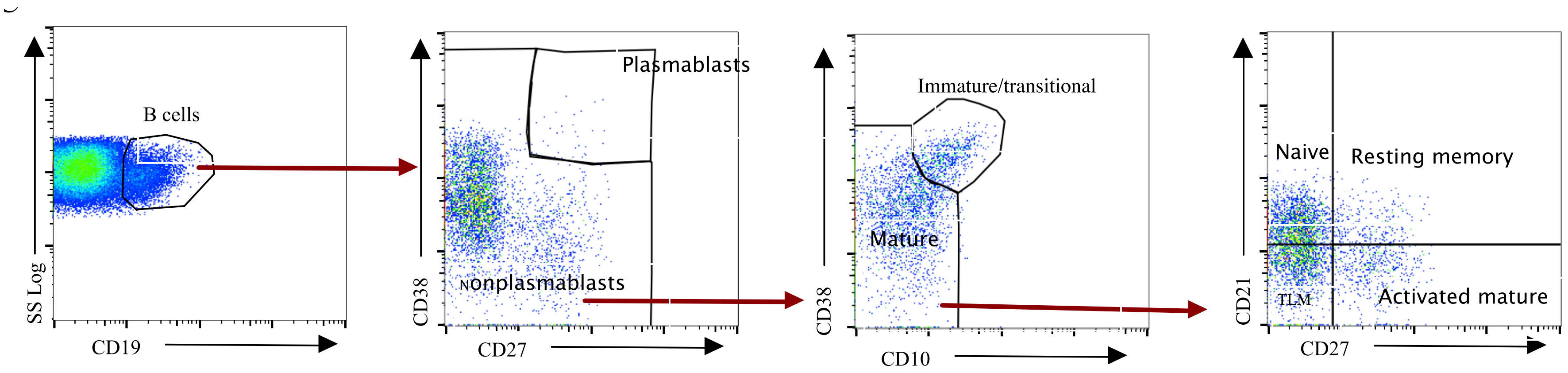

Supplement: S2 Fig — (PDF) [file pone.0175570.s003.pdf]
